# The First Evaluation of Workers’ General Health Examination in Korea

**DOI:** 10.1186/s40557-017-0158-z

**Published:** 2017-02-09

**Authors:** Eun-A Kim

**Affiliations:** Occupational Safety and Health Search Institute, Korea Occupational Safety and Health Agency, Ulsan, Republic of Korea

Workers health examination was introduced as an employers’ duty, in 1953, when the labor standard act was enacted. Since then, workers health examination have been one of the most important occupational health service to protect and promote workers’ health. It went to two different system in 1973, one is the workers’ special health examination (WSHE), which covered who is exposed to specific health risks at work, and the other is workers’ general health examination (WGHE), which covered who does not.

Since 1980, the Korean government started a health screening system for the general population. There has been several remodelings, including the schedule by age group, target diseases, and test items based on the comprehensive study, to evaluate the relevance and the effectiveness of the system [1]. Although workers’ health examination is one of the screening systems for the working population, it has never been evaluated for the effectiveness as a medical screening system, because the mission of workers’ health examination is not only medical screening, but it is expected to play a role as a health management tool. The necessity of the evaluation went unheard. At this moment, we did not known how the WGHE was successful, what the most important achievement of WGHE is, or whether we have to maintain this system.

In this special series, 4 papers draw the general outline, effectiveness, inequality and the practical use of the WGHE. These papers deal with the basic information and the evaluation of some key elements of WGHE that are essential to value the system, which might be a meaningful start of the evaluation and revision of workers’ health examination systems. Further, a detailed analysis regarding workers’ health examination must to be done.
